# Investigating the role of uncoupling of troponin I phosphorylation from changes in myofibrillar Ca^2+^-sensitivity in the pathogenesis of cardiomyopathy

**DOI:** 10.3389/fphys.2014.00315

**Published:** 2014-08-25

**Authors:** Andrew E. Messer, Steven B. Marston

**Affiliations:** National Heart & Lung Institute, Imperial College LondonLondon, UK

**Keywords:** troponin I, phosphorylation, cardiomyopathies, Ca sensitivity, heart muscle, myofilament

## Abstract

Contraction in the mammalian heart is controlled by the intracellular Ca^2+^ concentration as it is in all striated muscle, but the heart has an additional signaling system that comes into play to increase heart rate and cardiac output during exercise or stress. β-adrenergic stimulation of heart muscle cells leads to release of cyclic-AMP and the activation of protein kinase A which phosphorylates key proteins in the sarcolemma, sarcoplasmic reticulum and contractile apparatus. Troponin I (TnI) and Myosin Binding Protein C (MyBP-C) are the prime targets in the myofilaments. TnI phosphorylation lowers myofibrillar Ca^2+^-sensitivity and increases the speed of Ca^2+^-dissociation and relaxation (lusitropic effect). Recent studies have shown that this relationship between Ca^2+^-sensitivity and TnI phosphorylation may be unstable. In familial cardiomyopathies, both dilated and hypertrophic (DCM and HCM), a mutation in one of the proteins of the thin filament often results in the loss of the relationship (uncoupling) and blunting of the lusitropic response. For familial dilated cardiomyopathy in thin filament proteins it has been proposed that this uncoupling is causative of the phenotype. Uncoupling has also been found in human heart tissue from patients with hypertrophic obstructive cardiomyopathy as a secondary effect. Recently, it has been found that Ca^2+^-sensitizing drugs can promote uncoupling, whilst one Ca^2+^-desensitizing drug Epigallocatechin 3-Gallate (EGCG) can reverse uncoupling. We will discuss recent findings about the role of uncoupling in the development of cardiomyopathies and the molecular mechanism of the process.

## Introduction

The heart has a unique system for rapidly and precisely adjusting cardiac output to meet the demands put upon it. The rhythmic contraction and relaxation of heart muscle is due to the rise and fall of sarcoplasmic calcium ion (Ca^2+^) concentration under neural control. Contraction is initiated by Ca^2+^ release from the sarcoplasmic reticulum via the Ryanodine receptor and is terminated by Ca^2+^-uptake by the ATP-powered sarcoplasmic Ca^2+^ pump (SERCA). Ca^2+^ binds to troponin C (TnC), the Ca^2+^ receptor of the contractile apparatus to switch on contractile interactions between actin and the myosin motor protein in the thick filaments (Gordon et al., 2000; Macleod et al., 2010).

Independently of this Ca^2+^-switch, the speed and force of contraction is modulated in a graded way by changing the inotropic state of muscle. The inotropic state is largely controlled by the sympathetic system that releases β-adrenergic agonists at nerve endings and into the circulation from the adrenal glands. These bind to and activate β1 receptors on the cardiomyocyte surface and initiate a cascade leading to increased intracellular cyclic AMP concentrations which in turn activate the cyclic AMP dependent protein kinase (PKA) (Macleod et al., 2010). PKA phosphorylates several proteins in the sarcolemma, sarcoplasmic reticulum and the contractile apparatus, thus regulating their activity. The combined result of the action of PKA is a co-ordinated increase in cardiac output due to increased heart rate (chronotropy) increased force of contraction (inotropy) and increased rate of relaxation (lusitropy) (Layland et al., 2005).

PKA phosphorylates Myosin Binding Protein-C (MyBP-C) and troponin I (TnI) within the cardiac myofibril. TnI is the inhibitory component of the trimeric troponin molecule that makes up the Ca^2+^-switch of the contractile apparatus. TnI binds to TnC when Ca^2+^ is bound to TnC, whilst in the absence of Ca^2+^, the C-terminus of TnI is released and is able to interact with actin and tropomyosin to inhibit the thin filament's interaction with the motor protein, myosin. Thus, the interactions of TnC with TnI and Ca^2+^ are crucial for the Ca^2+^ control of muscle. Early studies showed that β-adrenergic stimulation of contraction was associated with enhanced phosphorylation of TnI (England, 1976; Solaro et al., 1976), that TnI was bis-phosphorylated at serines 22 and 23 in the cardiac-specific N-terminal extension by PKA (Mittmann et al., 1990; Al-Hillawi et al., 1995; Ayaz-Guner et al., 2009) and that the primary effect of phosphorylation of TnI *in vitro* was reduced Ca^2+^-sensitivity and faster dissociation of Ca^2+^ from TnC (Solaro et al., 1976; Robertson et al., 1982; Zhang et al., 1995; Dong et al., 2007). This can cause an increase in the rate of relaxation (lusitropic response) which is essential when heart rate is increased (Kentish et al., 2001; Layland et al., 2005). Transgenic mouse studies have demonstrated the physiological importance of TnI phosphorylation since mice with unphosphorylatable TnI have a blunted response to β-adrenergic stimulation and this leads to an enhanced susceptibility to the development of heart failure under stress (Fentzke et al., 1999; Pi et al., 2002; Yasuda et al., 2007).

Over the last 10 years it has become evident that the modulation of myofilament Ca^2+^-sensitivity by TnI phosphorylation is quite a labile system and that mutations associated with cardiomyopathies in particular, can lead to disruption of the system. This was first noted with mutations in TnI that caused hypertrophic cardiomyopathy (HCM) (Deng et al., 2001, 2003) but its physiological significance was uncovered by studies on dilated cardiomyopathy (DCM) (Dyer et al., 2007, 2009; Memo et al., 2013). DCM is a major cause of heart failure in humans and a substantial proportion of cases of DCM are inherited. Mutations in the thin filament proteins [actin, tropomyosin, troponin T (TnT), TnI, and TnC] that are associated with familial DCM have been studied particularly closely (reviewed in Chang and Potter, 2005; Morimoto, 2008; Marston, 2011). By studying isolated thin filaments with the quantitative *in vitro* motility assay (IVMA) it was found that in all of these DCM-causing mutations the myofilament Ca^2+^-sensitivity is independent of the level of TnI phosphorylation. Therefore, by analogy with the S22/23A transgenic mice, it was proposed that this uncoupling was necessary and sufficient to cause the DCM phenotype (Memo et al., 2013).

In this review we show that “uncoupling” of TnI phosphorylation from changes in Ca^2+^-sensitivity is a widespread phenomenon with significant implications for the understanding of heart disease and its treatment.

## Methodology

### Phosphorylation measurement

**Figure 1:**
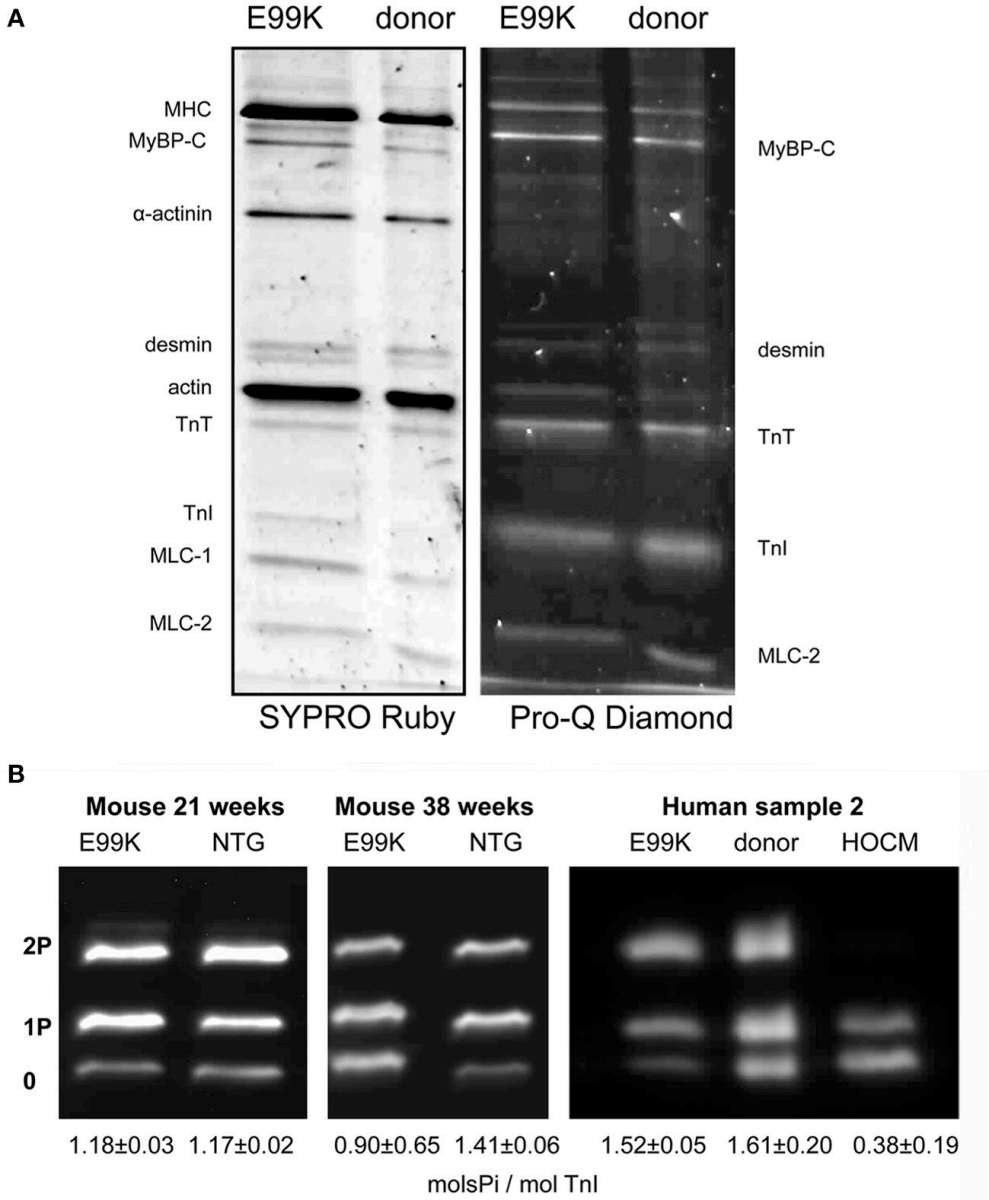
**SDS-PAGE analysis of myofibrillar proteins and phosphoproteins. (A)** Myofibril fraction of heart muscle separated by SDS-PAGE and stained successively with Pro-Q Diamond phosphoprotein stain and SYPRO Ruby total protein stain. Left, NTG and ACTC E99K mouse heart myofibrils (38 weeks male). Right, donor and ACTC E99K heart sample 2 myofibrils (Song et al., 2011). The TnI band is strongly stained together with MyBP-C, TnT and MLC-2. **(B)** Phosphate affinity SDS-PAGE analysis of TnI phospho-species. Left, comparison of TnI phosphorylation in 21-week-old female ACTC E99K and NTG mouse myofibrils. Right, comparison of TnI phosphorylation in myofibrils from ACTC E99K sample 2, donor heart and a typical interventricular septum sample from a patient with hypertrophic obstructive cardiomyopathy (HOCM) (Song et al., 2011). All samples show a high level of phosphorylation, except for the failing heart sample.

As there is a link between troponin (Tn) phosphorylation and Ca^2+^-sensitivity in cardiac muscle, measurement of troponin I (TnI) phosphorylation levels *in situ* is very important. Quantitative methods such as Top-down mass spectrometry and phosphate affinity SDS-PAGE has clearly established that serines 22 and 23 are the main amino acids phosphorylated in native heart tissue in rats, mice or humans (Zabrouskov et al., 2008; Ayaz-Guner et al., 2009; Marston and Walker, 2009; Messer et al., 2009; Sancho Solis et al., 2009; Wang et al., 2012) (these are often numbered 23 and 24 according to the coding sequence, however the N terminal methionine is missing in all mature TnI in heart tissue). The first quantitative studies used non-equilibrium pH gradient electrophoresis in 1 or 2D (Ardelt et al., 1998; Kobayashi et al., 2005). Measurement of phosphorylation became much easier with the introduction of specific methods to detect phosphoproteins using the phosphoprotein gel stain, Pro-Q Diamond (Steinberg et al., 2003) or antibodies to phosphorylated Tn (Al-Hillawi et al., 1998; Haworth et al., 2004). This methodology has been widely adopted, but has its limitations, since to be quantitative it requires the use of an external standard, which may introduce systematic errors (Figure 1A) (Messer et al., 2007; Zaremba et al., 2007).

Thus, to overcome this, we developed the use of phosphate affinity SDS-PAGE which was first developed by the Kinoshita group (Kinoshita et al., 2006). Phos-Tags are Mn^2+^-dependent specific chelators of phosphoproteins, when added to standard SDS-PAGE, phosphoproteins are retarded in proportion to the number of phosphates per mole of protein (Messer et al., 2009). Thus, unphosphorylated, monophosphorylated and bisphosphorylated species of phosphoproteins can be separated (Figure 1B). We have used Phos-Tags in conjunction with a specific cardiac TnI antibody to accurately measure phosphorylation levels in myofibrillar extracts from human heart tissue. The advantage of phosphate affinity SDS-PAGE is that it permits rapid identification and direct quantification of the mono and bis-phosphorylated TnI.

All the methods for measuring Ser22/23 phosphorylation in intact tissue give similar results: flash-frozen mouse or rat heart yield a phosphorylation level of 1–1.5 mol Pi/ mol TnI with up to 40% of TnI being the bis-phosphorylated species, whilst human donor heart samples have 1.5–2 mols Pi/mol TnI with up to 60% of bis-phosphorylated species. These types of samples have been widely used in the study of the role of TnI phosphorylation in modulating muscle regulation, however there is still controversy as to whether these samples are actually representative of the “normal” heart (Jweied et al., 2007; Marston and Detombe, 2008).

In contrast, pathological samples from hearts transplanted for idiopathic dilated cardiomyopathy or septal myectomies from patients with hypertrophic obstructive cardiomyopathy (HOCM) generally have a low level of phosphorylation (0.1–0.4 mols Pi/mol TnI) (Van Der Velden et al., 2003; Messer et al., 2007, 2009; Zaremba et al., 2007; Ayaz-Guner et al., 2009; Hamdani et al., 2009; Jacques et al., 2009; Bayliss et al., 2012b).

### Manipulation of TnI phosphorylation levels

**Figure 2:**
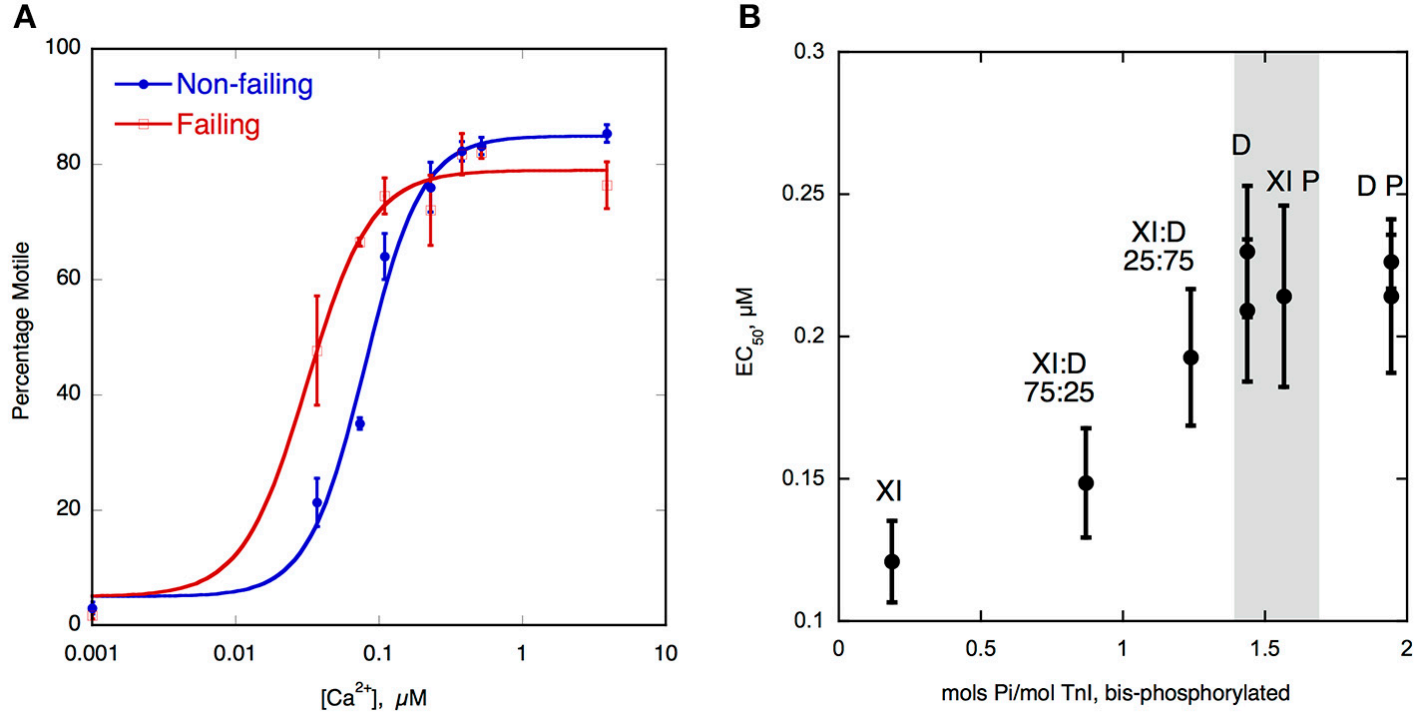
**(A)** Ca^2+^-regulation of thin filament motility by non-failing (donor) and failing heart troponin. Thin filament motility was measured by motility assay over a range of [Ca^2+^] in paired cells. The percentage of filaments motile is plotted as a function of [Ca^2+^] for a representative experiment. In blue lines and points, non-failing thin filaments, red lines and open points, failing thin filaments. TnI phosphorylation levels are shown in Figure 1B. The points ± s.e.m. are the mean of four determinations of percentage motile measured in one motility cell. The curves are fits of the data to the Hill equation (Messer et al., 2014). **(B)** Relationship between EC_50_ for Ca^2+^-activation of thin filaments and TnI bisphosphorylation. EC_50_ for thin filaments containing human heart Tn was plotted against the level of TnI bis-phosphorylation. EC_50_ was measured by IVMA and bis-phosphorylation of TnI (approximates to phosphorylation at Ser22 and 23) was measured by phosphate affinity SDS-PAGE. D, donor heart Tn, XI donor Tn with TnI exchanged, XI:D mixed donor and cTnI-exchanged donor, XI P PKA-treated cTnI-exchanged donor, D P PKA treated donor heart Tn. The gray band corresponds to the phosphorylation level range of human donor and wild-type mouse Tn, see Figure 1B (Memo et al., 2013).

To investigate the relationship between TnI phosphorylation and myofilament Ca^2+^-sensitivity, the Ca^2+^-sensitivity needs to be compared with phosphorylated and unphosphorylated Tn, thus the phosphorylation levels need to be manipulated. Initial *in vitro* work used Tn reconstituted from recombinant subunits expressed in E.coli; TnI could then be readily phosphorylated with PKA catalytic subunit. For transgenic mouse studies, unphosphorylatable TnI could be overexpressed (either slow skeletal TnI in place of cardiac or mutant TnI with Ser 22/23 mutated to alanine Fentzke et al., 1999; Pi et al., 2002; Yasuda et al., 2007). Phosphorylated TnI could be simulated with Ser 22/23 mutated to aspartic acid (Dohet et al., 1995; Mamidi et al., 2012). The first studies of native human heart Tn in IVMA or skinned myocyte contractility compared donor and failing human heart muscle samples, since they had high and low levels of phosphorylation respectively (Van Der Velden et al., 2003; Messer et al., 2007). However, it was not certain whether differences in Ca^2+^-sensitivity observed (Figure 2A) were due to the different phosphorylation levels or other disease-related factors.

Ideally, one should be able to study the same sample at different phosphorylation levels. This can be done by dephosphorylation or phosphorylation. The phosphorylation level of Tn isolated from heart tissue may be reduced by treatment with a phosphatase (shrimp alkaline phosphatase has proved to be the most reliable enzyme) or increased by PKA treatment (Bayliss et al., 2012b). PKA treatment has been used successfully for many years to increase the level of phosphorylation of isolated myocytes or skinned muscle strips (Hamdani et al., 2009; Kooij et al., 2010) but dephosphorylation is not usually successful, either there is inadequate reduction in phosphorylation level or the enzyme preparations cause degradation of the muscle. To dephosphorylate heart muscle in laboratory animals a different method may be used. For instance, mice can be treated with Propranolol (8 mg/kg) to block β1-adrenoreceptors and deactivate PKA to reduce phosphorylation levels of PKA substrates including Tn and MyBP-C (Bailin, 1979; Wang et al., 2011; Vikhorev et al., 2013).

## Normal relationship between TnI phosphorylation and Ca^2+^-regulation

It was established, soon after the discovery of troponin I (TnI) phosphorylation, that phosphorylation of troponin (Tn) modulates Ca^2+^-regulation by Tn by reducing the Ca^2+^-sensitivity and increasing the force or crossbridge turnover rate at maximally activating Ca^2+^ concentrations (Ray and England, 1976; Bailin, 1979; Mope et al., 1980). The magnitude of the Ca^2+^-sensitivity shift has been consistently been measured in the 2–3-fold range. It has been demonstrated that the reduced Ca^2+^-sensitivity is due to an increase in the rate of Ca^2+^-dissociation from Tn in the thin filaments (Robertson et al., 1982; Zhang et al., 1995; Dong et al., 2007), thereby providing a mechanism for the lusitropic (faster relaxation) response to β-adrenergic stimulation. Two recent studies have directly shown the relationship between phosphorylation and Ca^2+^-sensitivity. A study by Messer et al. used the *in vitro* motility assay (IVMA) with isolated human cardiac Tn in reconstituted thin filaments. By manipulating the level of Tn phosphorylation and then measuring the level using phosphate affinity SDS-PAGE (Phos-Tags) (Messer et al., 2009), a consistent relationship between phosphorylation level and Ca^2+^-sensitivity was found (Memo et al., 2013) (Figure 2B). A similar study by Kooij et al. measured the force in individual cardiomyocytes and found a similar relationship between TnI phosphorylation at Ser22/23 and Ca^2+^-sensitivity (Kooij et al., 2010). A reduced level of cTnI phosphorylation has been observed in end-stage failing hearts and this correlates with the increased Ca^2+^-sensitivity seen when failing hearts were compared to donor hearts (Figure 2A) (Wolff et al., 1995; Bodor et al., 1997; Van Der Velden et al., 2003; Messer et al., 2007).

## The discovery of uncoupling in familial cardiomyopathies

Initial investigations into the functional consequences of cardiomyopathy mutations did not consider the role of TnI phosphorylation, but when this was investigated, uncoupling was immediately apparent. Uncoupling was first reported in a series of studies from Kornelia Jaquet's laboratory. Deng et al. studied the cTnI HCM mutation R145G and compared phosphorylated and unphosphorylated recombinant R145G mutant Tn in reconstituted thin filaments regulating actomyosin ATPase. The authors found that the shift in pCa_50_ due to bisphosphorylation, observed with wild-type Tn, was not statistically significant (Deng et al., 2001). Later, two other HCM-causing mutations in cTnI, G203S, and K206Q, were also shown to uncouple, although the effect with G203S was only partial (Deng et al., 2003). A study on the cTnI HCM mutation R21C found that the Ca^2+^-sensitivity decrease due to PKA phosphorylation was smaller when compared to wild-type (Gomes et al., 2005). A similar study on the cTnC HCM mutation L29Q also found that the Ca^2+^-sensitivity (measured by both actomyosin ATPase activity and IVMA) was not affected by PKA phosphorylation of cTnI (Schmidtmann et al., 2005). In fact, this study was the first to suggest that the mutation hindered the transduction of the phosphorylation signal from TnI to TnC. Dong et al. have investigated the effects of cTnI phosphorylation on the kinetics of Ca^2+^ regulation of Tn both in wild-type and mutant Tn (Dong et al., 2007). The authors not only looked at the HCM-causing L29Q mutation in TnC but also the DCM-causing mutation TnC G159D and found that both mutations inhibited the ability of PKA phosphorylation of cTnI to reduce Ca^2+^-sensitivity and speed up Ca^2+^ dissociation (Dong et al., 2008).

The DCM-causing TnC G159D mutation is one of the best characterized clinically (Mogensen et al., 2004; Kaski et al., 2007) and the uncoupling phenomenon was also investigated in two further studies. Biesiadecki et al. reported that the cTnC G159D mutation, exchanged into skinned mouse cardiac fibers, had no direct effect on the myofilament response to Ca^2+^ but it blunted the phosphorylation-dependent change in Ca^2+^ sensitive tension development without altering cross-bridge cycling rate (Biesiadecki et al., 2007). Dyer et al., investigated Tn containing the TnC G159D mutation in mutant Tn isolated from an explanted heart muscle sample in comparison with donor heart Tn using IVMA and similarly found that, unlike donor heart, Ca^2+^-sensitivity and maximum sliding speed of thin filaments containing G159D Tn were not sensitive to changes in TnI phosphorylation levels (Dyer et al., 2007, 2009).

These seminal studies on uncoupling investigated mutations in the regions of TnI and TnC that could be directly involved in the phosphorylation-dependent interaction that modulates Ca^2+^-sensitivity. However, subsequent studies showed that mutations in any protein of the thin filament can induce uncoupling, including actin (ACTC E361G and E99K mutations) tropomyosin (E40K, E54K, and D230N mutations) and troponin T (TnT) (4 mutations recorded to date) in addition to 5 mutations in cTnC and 5 mutations in TnI. The currently known mutations causing uncoupling are summarized in Table 1. Thus, uncoupling may be induced by indirect allosteric effects of mutations anywhere within the thin filament and uncoupling seems to be correlated with mutations identified as causing cardiomyopathies.

**Table 1:** **Mutations that have been reported to cause uncoupling**.

**Mutation**	**Effect of mutation on Ca^2+^-sensitivity**	**Measurement method**	**Publication**
**DCM**			
ACTC E361G	No difference	IVMA	Song et al., 2010; Memo et al., 2013
TPM1 E54K	No difference	IVMA	Memo et al., 2013
TPM1 E40K	Decrease	IVMA	Memo et al., 2013
TPM1 D230N	Increase	IVMA	Memo et al., 2013
TNNC1 G159D	Increase	IVMA/Ca binding	Biesiadecki et al., 2007; Dong et al., 2008; Dyer et al., 2009; Memo et al., 2013
TNNC1 Y5H	Decrease	Skinned fiber	Pinto et al., 2011
TNNC1 M103I	Decrease	Skinned fiber	Pinto et al., 2011
TNNC1 I148V	Decrease	Skinned fiber	Pinto et al., 2011
TNNT2 ΔK210	Decrease	IVMA/Skinned fiber	Du et al., 2007; Inoue et al., 2013; Memo et al., 2013
TNNT2 R141W	Decrease	IVMA	Memo et al., 2013
TNNI3 K36Q	Decrease	IVMA/ATPase	Carballo et al., 2009; Memo et al., 2013
**HCM**			
ACTC E99K	Increase	IVMA	Song et al., 2011
TPM1 E180G	Increase	Skinned fiber	Alves et al., 2014
TNNC1 L29Q	Increase	Ca binding/ATPase	Schmidtmann et al., 2005; Dong et al., 2008; Li et al., 2013
TNNT2 K280N	Increase	IVMA	Bayliss et al., 2012a
TNNI3 R145G	Increase	IVMA/ATPase	Deng et al., 2001
TNNI3 R21C	Increase	Skinned fiber	Gomes et al., 2005; Wang et al., 2011
TNNI3 G203S	Increase	ATPase/IVMA	Deng et al., 2003
TNNI3 K206Q	Increase	ATPase/IVMA	Deng et al., 2003
TNNT2 R92W	Increase	Cardiac myocytes	Guinto et al., 2009

## Uncoupling as a primary cause of familial dilated cardiomyopathy

**Figure 3:**
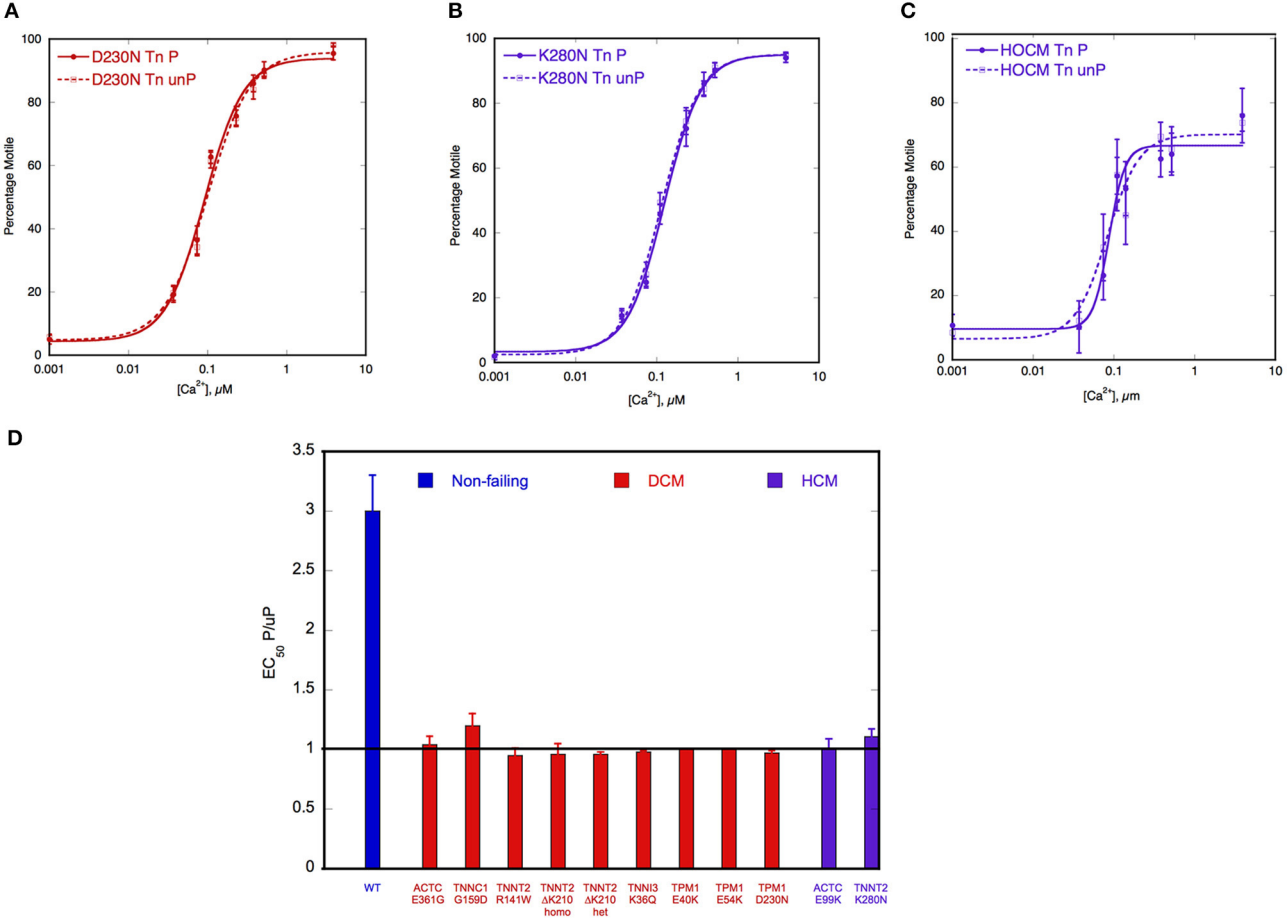
**Demonstration of uncoupling due to DCM and HCM mutations and secondary to HOCM**. Thin filament motility was measured by motility assay over a range of [Ca^2+^] in paired cells. The percentage of filaments motile is plotted as a function of [Ca^2+^] for a representative experiment. Solid lines and points, phosphorylated thin filaments, dotted line and open points, unphosphorylated thin filaments (obtained by phosphatase treatment). The points ± s.e.m. are the mean of four determinations of percentage motile measured in one motility cell. The curves are fits of the data to the Hill equation. **(A)** Natively phosphorylated and unphosphorylated human donor heart Tn with DCM related α- tropomyosin D230N (100%) and rabbit skeletal muscle α-actin (Memo et al., 2013). **(B)** Natively phosphorylated and unphosphorylated human donor heart Tn with exchanged recombinant HCM related TnT K280N, human heart tropomyosin and rabbit skeletal muscle α-actin (Messer et al., 2014). **(C)** Thin filaments containing Tn from a HOCM heart (0.1 mol Pi/mol TnI) or protein kinase A-treated HOCM heart Tn (1.6 mol Pi/mol TnI) (Bayliss et al., 2012b). **(D)** EC_50_ of phosphorylated Tn relative to EC_50_ of unphosphorylated Tn is plotted for wild-type thin filaments and thin filaments containing DCM and HCM-causing mutations. Error bars show s.e.m. of up to 7 replicate comparative measurements (Memo et al., 2013; Messer et al., 2014).

The recent study by Memo et al. (2013) investigated the uncoupling phenomenon in thin filaments containing a wide range of mutations associated with familial DCM, using the IVMA to measure myofilament Ca^2+^-sensitivity. It was found that when TnI was fully phosphorylated, the mutations had different effects on Ca^2+^-sensitivity of thin filaments compared to non-failing; some increased Ca^2+^-sensitivity (cTnT R141W and ΔK210, cTnI3 K36Q and α-Tropomyosin E40K), some decreased it (α-Tropomyosin D230N, cTnC G159D) whereas for α-actin E361G and α-Tropomyosin E54K there was no change in Ca^2+^-sensitivity. This confirmed that the simple hypothesis that Ca^2+^-sensitivity is always reduced by DCM-causing mutations, that we and others had proposed, is no longer tenable (Chang and Potter, 2005; Mirza et al., 2005; Morimoto, 2008). In contrast, when the Ca^2+^-sensitivity of thin filaments containing phosphorylated and unphosphorylated TnI were compared, it was found that incorporation of any of these mutations caused uncoupling (Memo et al., 2013) (Figure 3).

Another study found uncoupling in rare TnC variants identified in DCM: cTnC Y5H, M103I, and I148V either decreased or abolished the effects of PKA phosphorylation on Ca^2+^-sensitivity (Pinto et al., 2011). Since all the known DCM-causing mutations in thin filament proteins have now been shown to cause uncoupling, whilst having a very variable effect on absolute Ca^2+^-sensitivity and no DCM mutation has been demonstrated to have normal coupling, there is a strong case for uncoupling to be causative of DCM due to mutations of thin filament components. Thus, a blunting of the heart's response to β-adrenergic stimulation seems to be necessary and sufficient to generate the DCM phenotype. Mechanisms and physiological consequences of uncoupling are discussed in detail later in this review.

## Uncoupling is a common feature of cardiomyopathies

Uncoupling is widespread, not only is it observed with mutations that cause familial DCM, it is also observed with mutations that cause hypertrophic cardiomyopathy (HCM). The HCM phenotype has been generally thought to be due to mutations increasing myofilament Ca^2+^-sensitivity, but it is possible that uncoupling is also characteristic of HCM (Marston, 2011). In addition to the early reports of HCM-causing TnI mutations, described above, uncoupling has also been demonstrated in cTnT R92W (Guinto et al., 2009), cTnT K280N (Messer et al., 2012), α-Tropomyosin E180G (Alves et al., 2014), cardiac actin E99K (Song et al., 2011) and cTnI R21C (Wang et al., 2011) (see Table 1). The situation is less clear-cut in studies using human heart samples; Sequeira et al. (2013) reported that some HCM mutations were uncoupled, but others showed a partial decrease in Ca^2+^-sensitivity when phosphorylated indicating that uncoupling is not necessarily an all-or-nothing effect in human heart but may be graded. Relevant to these observations is the report that in human heart samples the effect of phosphorylation on EC_50_ was dependent upon background phosphorylation levels of other myofilament proteins (Kooij et al., 2010).

Uncoupling has been demonstrated to occur as a secondary effect unrelated to any mutation. In the obstructive variant of HCM (HOCM) the hypertrophied interventricular septum causes LVOTO (left ventricular outflow tract obstruction) and pressure overload. Several abnormalities in the contractile proteins in septal tissue from HOCM patients have been observed including; low phosphorylation levels of TnI and MyBP-C (Messer et al., 2009; Copeland et al., 2010b), differences in actin isoform expression (Copeland et al., 2010a) and loss of function in myosin (Jacques et al., 2008). Most of these abnormalities are shared with end-stage failing heart, but the Ca^2+^-sensitivity of Tn from HOCM samples, studied by IVMA, was not as expected from its low TnI phosphorylation level and this was found to be due to uncoupling of the relationship between Ca^2+^-sensitivity and TnI phosphorylation (Gallon et al., 2007; Jacques et al., 2008; Bayliss et al., 2012b). Uncoupling was demonstrated directly by comparing HOCM Tn with PKA-treated HOCM Tn to bring phosphorylation up to the same level as donor heart Tn. There was no change in Ca^2+^-sensitivity (Figures 3C,D). This uncoupling was independent of the mutation causing HCM and was even observed when no mutation was identified. Exchange experiments were carried out to identify which component of the Tn complex was responsible for the uncoupling and the abnormality was shown to be in TnT, although no covalent modifications were found (Bayliss et al., 2012b). This uncoupling in HOCM may be related to the severe pressure overload that patients having myectomy operations exhibit and therefore it is possible that the uncoupling is caused by the pressure overload itself. It would, for instance, be interesting to look at aortic stenosis samples where the patients have pressure overload but not HCM (Marston et al., 2012).

The occurrence of uncoupling in other types of cardiomyopathy has not been tested; it is clear that in most cases of idiopathic DCM, Ca^2+^-sensitivity is fully coupled to the level of TnI phosphorylation (Messer et al., 2007). On the other hand, it is possible that mutations in sarcomeric proteins that are not part of the contractile apparatus, such as titin or Z-line proteins, also undergo uncoupling, since these can show a blunted response to β-adrenergic stimulation *in vitro* that is characteristic of uncoupling. Recent studies have shown a blunted β-adrenergic response in MLP W4R and TCAP KO mice (Knoell et al., 2010, 2011) and uncoupling could be inferred from experiments on a mouse model with a titin mutation (Gramlich et al., 2009).

## Uncoupling can be induced by small molecules and phosphorylation

**Figure 4:**
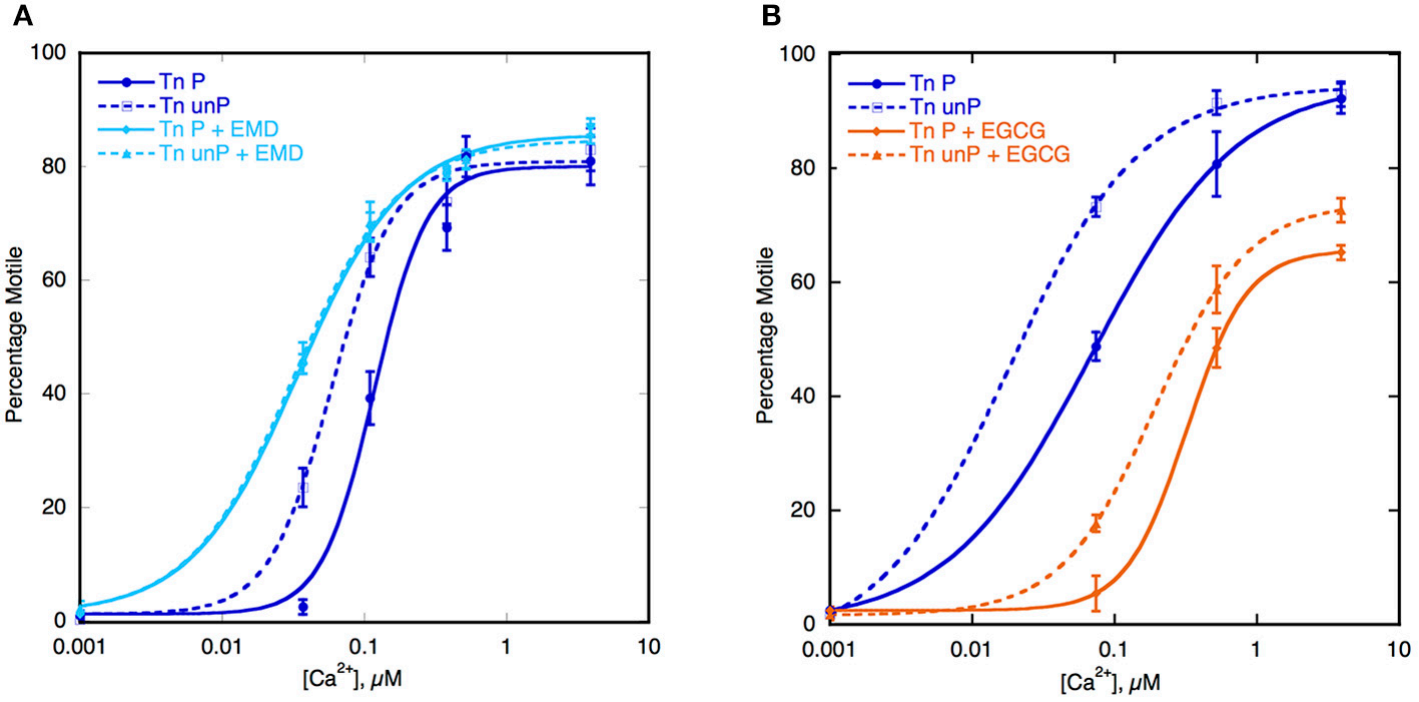
**Effect of Ca^2+^-sensitizers and desensitizers on coupling in thin filaments**. Thin filament motility was measured by motility assay over a range of [Ca^2+^] in paired cells. The percentage of filaments motile is plotted as a function of [Ca^2+^] for representative experiments. Solid lines and points, phosphorylated thin filaments, dotted line and open points, unphosphorylated thin filaments (obtained by phosphatase treatment). The points ± s.e.m. are the mean of four determinations of percentage motile measured in one motility cell. The curves are fits of the data to the Hill equation. **(A)** The effect of the Ca^2+^-sensitizer EMD57033 (EMD). In dark blue, thin filaments in the absence of EMD and in light blue are thin filaments in the presence of 30 μM EMD (Messer et al., 2014). **(B)** The effect of the Ca^2+^-desensitizer EGCG. In dark blue, thin filaments in the absence of EGCG and in orange, thin filaments in the presence of 100 μM EGCG (Messer et al., 2014).

The key to the modulation of Ca^2+^-sensitivity by cTnI phosphorylation is the interaction of the N-terminal peptide 1–29 of cTnI with TnC, therefore it may be possible to induce uncoupling with small molecules that bind to TnC and change the Ca^2+^-sensitivity (Ca^2+^ sensitizers or desensitizers). Of particular interest are the Ca^2+^-sensitizing drugs EMD57033 and Bepridil (Li et al., 2008). When tested in the IVMA, with thin filaments containing native human Tn, both these drugs increased Ca^2+^-sensitivity by 2–3-fold and at the same time uncoupled Ca^2+^-sensitivity from TnI phosphorylation (see Figure 4A). The effect of these drugs is therefore quite analogous to the effect of many HCM-causing mutations (Table 1, Figures 3, 4).

In contrast EGCG [(−)-Epigallocatechin 3-Gallate], is a Ca^2+^-desensitizer that binds at a site formed by the TnI-TnC complex and it has been found to enhance the binding of the N terminal helix1 of TnI to TnC (Robertson et al., 2009; Tadano et al., 2010; Botten et al., 2013). When tested in the IVMA, with thin filaments containing native human Tn, EGCG decreased Ca^2+^-sensitivity both in wild-type and in DCM-mutant thin filaments and in both phosphorylated and unphosphorylated filaments, thus preserving coupling (Figure 4B). Most strikingly it is also capable of restoring coupling to thin filaments containing mutations that induce uncoupling (Messer et al., 2014).

Another perturbation that can induce uncoupling is phosphorylation of troponin subunits. A study by Nixon et al. found that phosphorylation of cTnI at Ser 150 by AMP-activated protein kinase (AMPK) increased Ca^2+^-sensitivity of isolated cardiac myofibrils. It also blunted the PKA-dependent calcium desensitization induced by phosphorylation at Ser 22/23 and uncoupled the effects of phosphorylation from β-adrenergic stimulation (Nixon et al., 2012).

## Molecular mechanism of uncoupling

The phosphorylation dependence of cardiac Tn Ca^2+^ regulation is due to the interaction of a 30 amino acid N-terminal extension of TnI, containing the PKA-specific phosphorylation sites at Ser 22 and 23, with cTnC (Solaro et al., 2008).

The N-terminal segment of cTnI interacts with the regulatory Ca^2+^-binding loop in the N-terminal lobe of TnC in the unphosphorylated state. This affects the cTnC interaction with both the regulatory Ca^2+^ and the TnI switch peptide (144–160) (Li et al., 2008). When TnI is unphosphorylated there is a weak ionic bond between the N terminal and the regulatory Ca^2+^-binding EF hand of TnC (Howarth et al., 2007). When Ser 22 and 23 are phosphorylated the binding is further weakened (Keane et al., 1990; Ferrieres et al., 2000; Ward et al., 2004a,b; Baryshnikova et al., 2008). Therefore, the unphosphorylated state of TnI is a special state, which is destabilized by phosphorylation, resulting in a lower Ca^2+^-sensitivity and higher rate of Ca^2+^ dissociation. Since the initial interaction is quite weak, the loss of the interaction produces only a 2–3-fold change in Ca^2+^-sensitivity and the rate of Ca^2+^ dissociation. This appears to be sufficient to generate the lusitropic effect since heart rate also increases by a maximum of 2–3-fold.

We propose that the unphosphorylated state can also be disrupted by mutations or other alterations in any component of the thin filament resulting in the same destabilized state for both phosphorylated and unphosphorylated Tn; in this way uncoupling could be considered as a default state in cardiomyopathies (Liu et al., 2012; Memo et al., 2013).

Recent studies have begun to determine the structure of TnI in complex with TnC in the phosphorylated and unphosphorylated states that forms the basis of the coupling mechanism. X-ray crystallography has defined the core structure of Tn but mobile segments, including the N-terminus of TnI, were not present in the crystal structure (Takeda et al., 2003). NMR studies have defined the structure of the missing peptides based on their binary complexes. A best guess structure of the N-terminal peptide conformation in the phosphorylated and unphosphorylated states was proposed by building these structures onto the Tn core structure (Howarth et al., 2007). Molecular dynamics simulations of the entire Tn molecule have further refined these structures.

**Figure 5:**
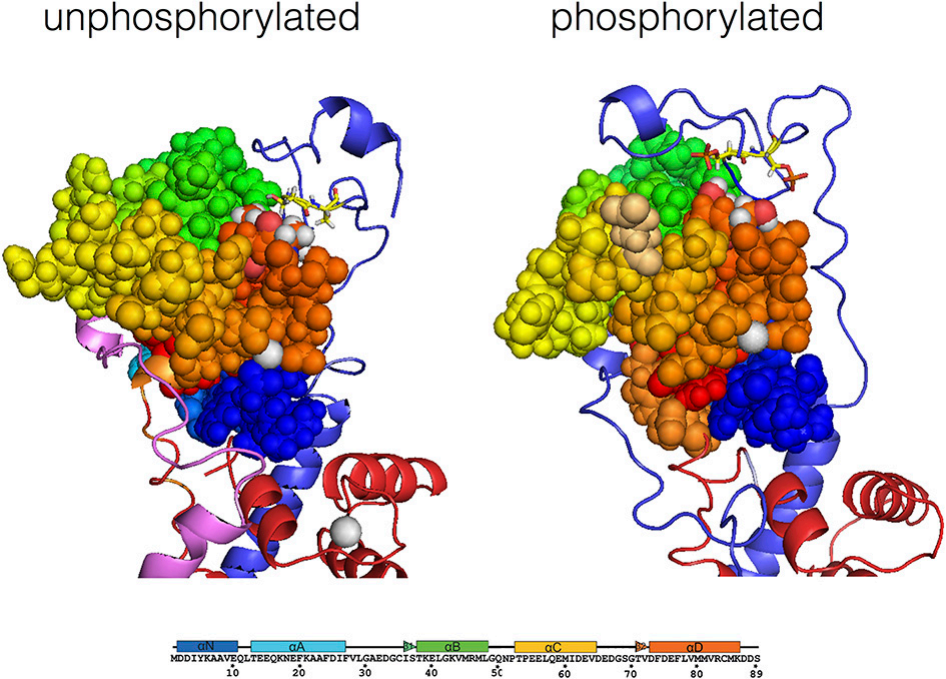
**Modulation of the interaction of TnI Ser 22 and 23 with the N terminal lobe of TnC by phosphorylation, determined by molecular dynamics simulations**. Average structure after 700 ns simulation for unphosphorylated Tn, left. The Tn was phosphorylated *in silico* after 550 ns of simulation and the average structure after a further 200 ns is shown, right. The N-terminal lobe of cTnC is shown as spheres with color coding along the peptide chain as indicated by the sequence below; Ser 69 and Thr 71 are colored according to their atoms (oxygen red, hydrogen white). The backbone of cTnI N-terminus is shown in blue; Ser 22 and 23 are shown in yellow in stick representation. Molecular dynamics suggests that with unphosphorylated Tn there is a close interaction of Ser 22 and 23 with cTnC Ser 69 and Thr 71 which is much less prominent when phosphorylated (Gould et al., 2014).

The molecular dynamics simulations indicate a possible structure of TnI N-terminus interacting with TnC (Gould et al., 2014) (Figure 5). The most striking feature is that in the presence of Ca^2+^, the unphosphorylated N terminus of TnI settles in a position looping over the N-terminus of TnC within 50 ns of the start of simulation. The peptide is mostly very mobile and unstructured except for ^20^RRSS^24^ that was consistently close to TnC for up to 1 μs of simulation. These four amino acids also exhibited a lower root mean square fluctuation (RMSF) than surrounding residues. When the Ser 22 and 23 were phosphorylated *in silico*, the two serines become more mobile relative to arginines 20 and 21 suggesting a weakening of their interaction with TnC. In addition, Ca^2+^ becomes more exposed to solvent and the interaction of the “switch peptide” with TnC is altered. Thus, coupling can be accounted for by the formation of a weak ionic complex between TnC and TnI Ser 22 and 23 that is destabilized by phosphorylation.

It is interesting to note that when the DCM-causing mutation K36Q in cTnI was introduced in the presence of Ca^2+^, the simulation showed that Ser 22 and 23 no longer interacted closely with cTnC, in accord with our hypothesis that the Ca^2+^-cTnC-cTnI N terminus interaction is unique and is destabilized directly by phosphorylation and also allosterically by mutations and other perturbations. Molecular dynamics simulations also show that phosphorylation is associated with long-range conformational changes in Tn and associated proteins that provides a mechanism for mutations in TnT, tropomyosin and actin to induce uncoupling (Manning et al., 2011). It should be noted that this mechanism for uncoupling is the opposite to one proposed by Biesiadecki et al. (2007) where the DCM mutation TnC G159D was proposed to *stabilize* the interaction of Ser 22 and 23 with cTnC when *phosphorylated*.

## Physiological relevance of uncoupling

How is uncoupling of the relationship between TnI phosphorylation and myofilament Ca^2+^-sensitivity related to the DCM phenotype associated with such mutations? We think it is likely that uncoupling would compromise the heart's response to β1-adrenergic stimulation leading to a reduced cardiac reserve.

**Figure 6:**
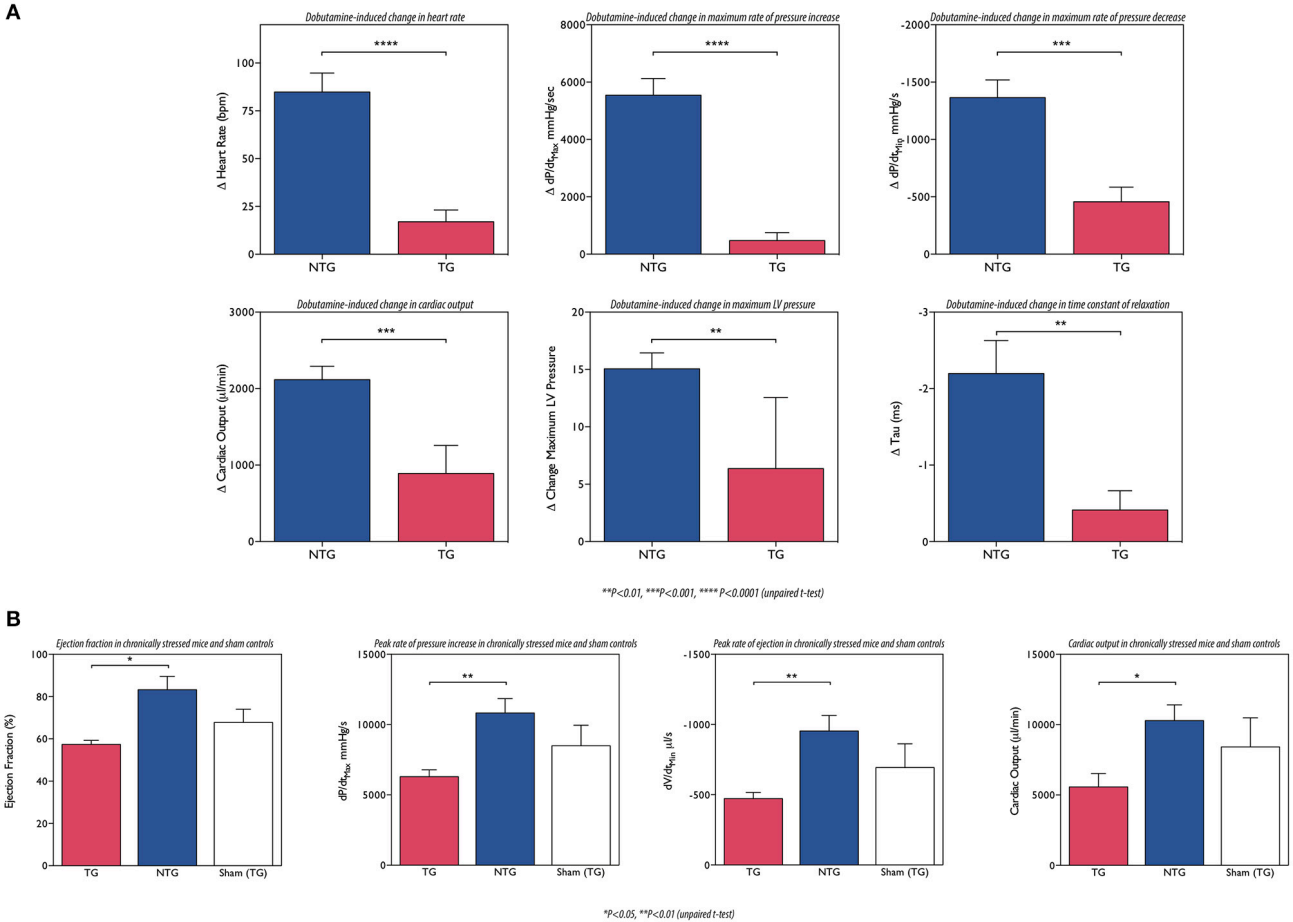
**(A)** The ACTC E361G mutation blunts the lusitropic, inotropic and chronotropic response to dobutamine *in vivo*: mice were examined using a pressure volume catheter. The dobutamine-induced acceleration of relaxation (peak rate of relaxation and time constant of relaxation) was significantly lower in ACTC E361G mice indicating a blunted lusitropic response. The inotropic response to dobutamine was also blunted in ACTC E361G mice as indicated by a blunted increase in maximum pressure and the peak rate of pressure increase. Furthermore, dobutamine-induced increase in heart rate (chronotropic effect) was also blunted. Taken together with the attenuated increase in cardiac output these data suggest a significantly diminished cardiac reserve in ACTC E361G mice *in vivo* (Wilkinson, 2014). **(B)** The ACTC E361G mutation predisposes TG mice to systolic heart failure under chronic stress: chronic stress was achieved using subcutaneously implanted Alzet osmotic mini pumps to deliver a 4-week infusion of angiotensin II (2 mg/kgBW/day, in saline). Sham controls received mini pumps carrying saline only. At the end of the 4-week infusion period the mice were examined using a pressure volume catheter. The chronic stress treatment evoked symptoms of systolic heart failure in ACTC E361G mice, characterized by decreased ejection fraction, cardiac output and maximum rates of contraction and ejection compared to NTG mice (Wilkinson, 2014).

The effects of cardiomyopathy-causing mutations on the heart's response to β-adrenergic agonists have not been routinely measured. However, the studies of Song et al. on the ACTC mutations E361G and E99K investigated this question and clearly showed that the response to dobutamine stimulation was blunted (Song et al., 2010, 2011; Marston et al., 2013) (Figure 6). The effect of adrenergic agonists was tested in several other models of HCM and DCM (see Table 2) and they all showed blunting of the response in at least one parameter. It is particularly interesting to note the blunting effect of the muscle LIM protein (MLP) W4R mutation associated with DCM (Knoell et al., 2010), since this protein is a component of the Z-line and is not known to have any function in regulating the contractile apparatus: in this case the putative uncoupling might be a secondary effect similar to that seen in myectomy samples. Nguyen et al. found that young, pre-hypertrophic *TNNI3* G203S HCM transgenic mice lacked the normal physiological response to chronic intense swimming exercise, compatible with a blunted response to adrenergic stimulation independent of disease phenotype (Nguyen et al., 2007).

**Table 2:** **Mutations reported to blunt response to β-agonists**.

**Mutation**	**Measurement method, agonist and (parameters blunted)**	**Publication**
ACTC E361G	Echocardiography and cine MRI Dobutamine stimulation (mri: EF, HR,COEcho: wall thickening and CO)	Song et al., 2010
ACTC E99K	Echocardiography Dobutamine stimulation (heart rate, wall thickening, and fractional shortening)	Song et al., 2011
TPM1 E54K	Echocardiography Isoprenaline stimulation (+dP/dt, −dP/dt)	Rajan et al., 2007
TNNT2 ΔK210	PV catheter Isoprenaline stimulation (LVESP)	Du et al., 2007
TNNT2 R173W	iPSC myocyte cluster contraction, noradrenaline stimulation (beating rate)	Sun et al., 2012
TNNT2 ΔE160	Isolated heart isovolumic pressure recording, Dobutamine stimulation (−dP/dt)	Moore et al., 2013
TNNT2 R92Q	Isolated heart isovolumic pressure recording, Dobutamine stimulation (SP, +dP/dt, −dP/dt, DevP)	Javadpour et al., 2003
TNNT2 I79N	Echocardiography, Isoprenaline stimulation (FS)	Knollmann et al., 2001
MYBPC3 KI	Engineered heart tissue Isoprenaline stimulation (ΔForce)	Stöhr et al., 2013
MYBPC3 KO	PV catheter Dobutamine stimulation (dP/dt_*max*_)	Carrier et al., 2004
MLP W4R	Echocardiography and PV catheter Adrenaline stimulation (ESV,EDV and LV contractility)	Knoell et al., 2010

This loss of cardiac reserve is likely to predispose the heart to failure when under stress. It is notable that most mouse models of DCM-causing mutations show little or no phenotype at rest (ACTC E361G, TNNT2 ΔK210, MYBPC3 knock-in (KI) (see Table 2) and TTN KI Gramlich et al., 2009), especially when heterozygous like the patients with these mutations. This is compatible with the primary defect being in the response to β-adrenergic stimulation that is absent at rest. Several experiments have addressed this question by exposing transgenic mice with HCM or DCM-causing mutations to chronic stress by pressure overload (TAC) or by chronic stimulation with isoprenaline or angiotensin II. In general, they demonstrate that the mutant-containing mice show earlier and more severe symptoms of dilation and heart failure than wild-type. For instance: Wilkinson applied chronic stress by angiotensin II infusion (2 mg/KgBV/da by osmotic minipumps). After 4 weeks *ACTC* E361G DCM mice had significantly lower dP/dtmax, cardiac output and ejection fraction, compared to NTG (Wilkinson, 2014) (Figure 6). Similarly Gramlich et al. studied a titin mutation that causes DCM (TTN 2-bp insertion mutation (c.43628insAT)) (Gramlich et al., 2009). The authors induced cardiac hypertrophy by a 2-week infusion with angiotensin II. Both wild-type and KI mice developed cardiac hypertrophy after 1 week. After 2 weeks, hypertrophy in wild-type animals was further increased, whereas their heterozygous littermates showed left ventricular dilatation with impaired systolic function and increased myocardial fibrosis.

Whilst it is recognized that the uncoupling phenomenon provides a satisfactory molecular mechanism for thin-filament based mutations that cause DCM, the role of uncoupling in HCM is not as clear. Since Ca^2+^-sensitivity has been observed to be increased 2–3-fold in virtually every HCM mutation investigated (Marston, 2011), it is likely that this is the primary trigger for the HCM phenotype and that it dominates over the uncoupling phenomenon. It is possible that increased Ca^2+^-sensitivity and uncoupling are linked properties of thin filaments since the Ca^2+^-sensitizers EMD57033 and bepridil are also uncouplers and the coupling constant is generally greatest when Ca^2+^-sensitivity is lowest. It will be very interesting to investigate whether any HCM mutations (or Ca^2+^-sensitizers) can be found that increase Ca^2+^-sensitivity but do not uncouple.

## Clinical relevance of uncoupling

Uncoupling inevitably leads to blunting of the response to β-adrenergic agonists but the lack of response to dobutamine is of course not only due to uncoupling. Heart failure is associated also with desensitization of β-receptors, such that the activation of PKA is attenuated, or the activity of phosphatase increased whilst coupling is intact (Houser and Margulies, 2003; Champion, 2005; El-Armouche et al., 2007; Messer et al., 2007).

Patient studies from the 80 and 90 s using echocardiography showed that IDCM and HCM patients could be classified into dobutamine responders and non-responders and that the non-responders have a poor prognosis whilst the responders can respond to treatment (Borow et al., 1988; Dubois-Randé et al., 1992; Naqvi et al., 1999) These studies predate the discovery of mutations in contractile proteins that cause familial DCM as well as the discovery of uncoupling, but given our current understanding of FDCM we would predict that the dobutamine non-responders correspond to those patients with FDCM mutations causing uncoupling and hence presumably the dobutamine response would be of clinical interest as a potential diagnostic to distinguish familial DCM from acquired IDCM.

This dichotomy would suggest that different treatments would be optimum for the two cases. Drugs are available that impact on β-receptors but so far no drugs act positively on the TnI-phosphorylation-Ca^2+^-sensitivity coupling mechanism. Our recent finding that EGCG is capable of recoupling *in vitro*, although it has different effects *in vivo* (Feng et al., 2012), suggests that specific modulation of the coupling process may be a viable target for future therapy.

### Conflict of interest statement

The authors declare that the research was conducted in the absence of any commercial or financial relationships that could be construed as a potential conflict of interest.
